# Draft Genome Sequence of *Tepidibacillus decaturensis* Strain Z9, an Anaerobic, Moderately Thermophilic, and Heterotrophic Bacterium from the Deep Subsurface of the Illinois Basin, USA

**DOI:** 10.1128/genomeA.00190-16

**Published:** 2016-04-07

**Authors:** Yiran Dong, Yun-Juan Chang, Robert A. Sanford, Bruce W. Fouke

**Affiliations:** a Energy Biosciences Institute, University of Illinois, Urbana-Champaign, Illinois, USA; b Department of Geology, University of Illinois, Urbana-Champaign, Illinois, USA; c Carl R. Woese Institute for Genomic Biology, University of Illinois, Urbana-Champaign, Illinois, USA; d Roy J. Carver Biotechnology Center, University of Illinois, Urbana-Champaign, Illinois, USA; e Department of Microbiology, University of Illinois, Urbana-Champaign, Illinois, USA; f High Performance and Research Computing, Office of Information Technology, Rutgers University, New Brunswick, New Jersey, USA

## Abstract

The genome of the moderately thermophilic and halotolerant bacterium *Tepidibacillus decaturensis* strain Z9 was sequenced. The draft genome comprises three scaffolds, for a total of 2.95 Mb. As the first sequenced genome within the genus *Tepidibacillus*, 2,895 protein-coding genes, 52 tRNA genes, and 3 rRNA operons were predicted.

## GENOME ANNOUNCEMENT

*Tepidibacillus decaturensis* strain Z9 was isolated from the groundwater from 1.7 km depth at the Cambrian Mt. Simon Sandstone of the Illinois Basin, Decatur, IL (1). This organism exhibits 97% identity in the 16S rRNA gene with its phylogenetically closest relative *Tepidibacillus fermentans*, an isolate obtained from an underground gas storage reservoir (2), and no higher than 95% identity with the other type strains affiliated with the family *Bacillaceae*. Strain Z9 was able to grow at salinities ranging 1 to 5% (NaCl), below the native salinity condition (15%). However, growth occurred at the range of temperatures 20 to 60°C and pH 5 to 8, which covered the native environmental conditions (temperature, 47°C; pH 6.31) (1). In addition to reducing nitrate to nitrite and fermenting different organic substrates, *T. decaturensis* strain Z9 actively reduces Fe(III)-citrate, ferrihydrite, lepidocrocite and other transition metals (e.g., MnO_2_, Cr_2_O_7_2^-^, and Co(III)-EDTA) using different organic and inorganic substrates as the electron donors (Y. Dong, R. A. Sanford, M. I. Boyanov, K. M. Kemner, T. M. Flynn, E. J. O’Loughlin, R. A. I. Locke, J. R. Weber, S. M. Egan, and B. W. Fouke, unpublished data). This organism represents a phylogenetically and physiologically unique population that inhabits deep subsurface and may play an important role in biogeochemical processes.

High-quality genomic DNA was sequenced with both the Roche GS-FLX sequencer and Illumina HiSeq 2000 sequencer. The draft genome was assembled using hybrid approach with Newbler (version 2.7). The draft assembly was then error corrected using eight iterations of iCORN 0.97 (3). The corrected sequences and the remaining Illumina reads were under internal gap closure with the aid of GapCloser for SOAP*denovo* (version 1.12) (4). The assembly generated three scaffolds with a total size of 2.95 Mb and a G+C content of 36.1%. Genome annotation was performed using the IMG-ER pipeline (5). A total of 2,895 protein-coding genes were predicted, including 3 sets of rRNA genes (5S rRNA, 16S rRNA, and 23S rRNA) and 52 tRNA genes.

Gene annotation indicated a broad nutrient diversity for strain Z9. The genome contains multiple pathways for carbon metabolism, including glycolysis, tricarboxylic acid (TCA) cycle, and pentose phosphate pathway (PPP), consistent with its capacity to ferment different mono- and polysaccharides (Dong et al., unpublished data). Genes coding for short-chain fatty acid metabolism reflected diversity in organic substrate utilization. This would support various terminal electron-accepting processes [e.g., nitrate and Fe(III) reduction] (Dong et al., unpublished data). The genome revealed the presence of nitrate-nitrite transporters (*narK*, *nasA*, or *nrtP*) and dissimilatory and assimilatory nitrate reductases (*narG*, *narH*, *narJ*, and *narI*), which is consistent with our observation that strain Z9 carried out nitrate reduction with nitrite as the product (Dong et al., unpublished data). In contrast to *T. fermentans*, which reduces sulfur and thiosulfate, strain Z9 lacks key genes involved in sulfur cycling (e.g., sulfate adenylyltransferase, adenylyl-sulfate reductase, and sulfite reductase). Strain Z9 contained *c*-type cytochromes, as has been shown with other known metal reducers (e.g., *Geobacter* and *Shewanella* species) (6), which may enable it to catalyze iron reduction.

### Nucleotide sequence accession numbers.

The whole-genome sequence of *T. decaturensis* strain Z9 has been deposited in NCBI GenBank under the accession no. LSKU00000000. The version described in this paper is LSKU01000000.
